# Supplementary material for: Endogenous transcripts control miRNA levels and activity in mammalian cells by target-directed miRNA degradation
Source: Nat Commun. 2018 Aug 7;9:3119. doi: 10.1038/s41467-018-05182-9 (PMC6081425; doi:10.1038/s41467-018-05182-9)
Supplement: Supplementary file 1 — Supplementary Information [file 41467_2018_5182_MOESM1_ESM.pdf]

Endogenous Transcripts Control miRNA Levels and Activity in Mammalian Cells by Target-Directed miRNA Degradation

Ghini *et al*.

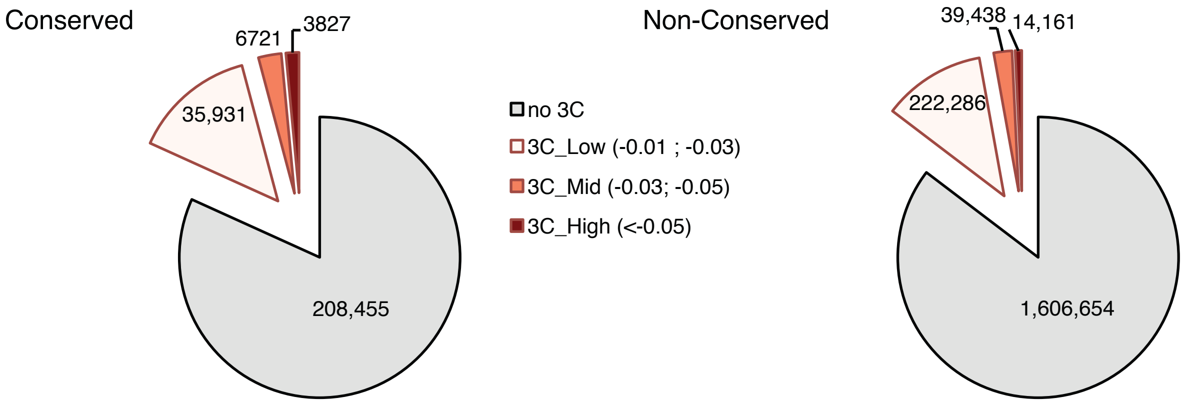

Supplementary Figure 1 - related to Figure1. Classification of mouse MREs according to 3' pairing score.

Pie charts show conserved (left chart) and non conserved miRNA responsive elements (MREs) present in the mouse genome according to TargetScan6.2 databases. MREs are classified in four groups based on the 3' pairing score (no, Low, Mid, High). Score intervals are shown in the legend.

a

ID transcript	Type	Seed match	3C-score	Name	15	30	45	60	90	120	240	360	480
					% of Total 3C-Target Pool during time course (minutes)								
NM_008871	CS	8mer	-0.081	*Serpine1	***1.18%***	***4.69%***	***10.23%***	***16.66%***	***29.70%***	***35.02%***	***29.89%***	***23.16%***	***19.74%***
NM_001145209	CS	8mer	-0.071	Hbs1l	0.79%	0.71%	0.63%	0.64%	0.53%	0.51%	0.56%	0.62%	0.75%
NM_144925	CS	7mer-m8	-0.062	Tnrc6a	0.24%	0.20%	0.19%	0.15%	0.13%	0.11%	0.11%	0.12%	0.13%
NM_001163703	CS	8mer	-0.060	Dcun1d3	0.09%	0.15%	0.20%	0.18%	0.11%	0.08%	0.07%	0.06%	0.06%
NM_013509	NCS	8mer	-0.060	Eno2	0.06%	0.04%	0.05%	0.04%	0.04%	0.04%	0.02%	0.03%	0.02%
NM_001081363	NCS	7mer-m8	-0.053	Cenpf	0.09%	0.07%	0.07%	0.08%	0.04%	0.04%	0.03%	0.03%	0.04%
NM_021284	CS	7mer-1A	-0.053	Kras	0.12%	0.12%	0.12%	0.14%	0.12%	0.19%	0.25%	0.21%	0.23%
NM_145629	NCS	7mer-m8	-0.053	*Pls3	***1.65%***	***1.37%***	***1.41%***	***1.43%***	***1.18%***	***1.44%***	***2.04%***	***2.31%***	***2.34%***
NM_175407	CS	7mer-m8	-0.053	Sobp	0.00%	0.00%	0.00%	0.00%	0.00%	0.00%	0.00%	0.00%	0.00%
NM_025630	NCS	8mer	-0.050	Aggf1	0.28%	0.25%	0.24%	0.23%	0.20%	0.23%	0.28%	0.27%	0.29%
NM_025958	NCS	8mer	-0.050	Cand2	0.06%	0.05%	0.05%	0.04%	0.04%	0.03%	0.03%	0.02%	0.02%
NM_001170691	CS	8mer	-0.050	Elmod2	0.18%	0.20%	0.17%	0.14%	0.11%	0.08%	0.06%	0.07%	0.07%
NM_028007	CS	8mer	-0.050	*Itfg1	***1.00%***	***1.11%***	0.97%	0.88%	0.73%	0.66%	0.55%	0.53%	0.38%
NM_001081173	CS	8mer	-0.050	Lrch2	0.08%	0.09%	0.07%	0.07%	0.04%	0.04%	0.03%	0.04%	0.05%
NM_001037221	CS	8mer	-0.050	Samd4	0.27%	0.26%	0.23%	0.22%	0.29%	0.39%	0.30%	0.25%	0.24%
NM_080448	CS	8mer	-0.050	Srgap3	0.08%	0.07%	0.07%	0.06%	0.04%	0.03%	0.01%	0.01%	0.01%

b

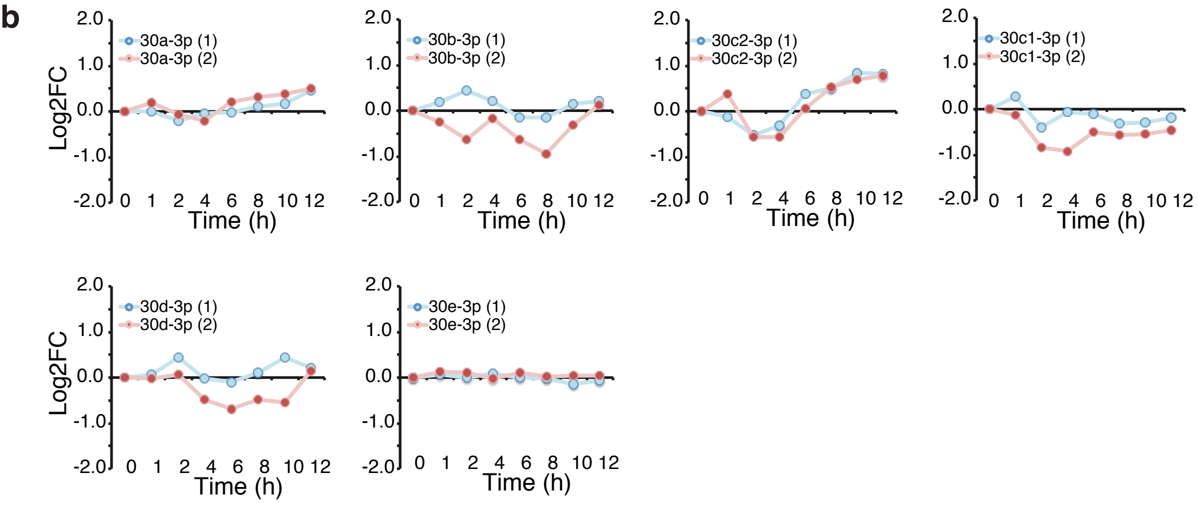

Supplementary Figure 2 - related to Figure 2. High-complementarity Targets for miR-30b/c and expression of miR-30 passenger miRNAs.
(**a**) Table shows details of all the targets for miR-30b/c with ‘high’ complementarity (3C-score <-0.05). Shown are the transcript ID, the conservation and the type of seed match, the 3C-score, the gene symbol together with the contribution (as percentage, %) over the total 3C-target pool calculated during the time course. Target reaching at least 1% are marked with an asterisks. (**b**) Expression of passenger miRNAs of the miR-30 family upon serum stimulation of quiescent 3T9 fibroblasts was measured by small RNA sequencing.

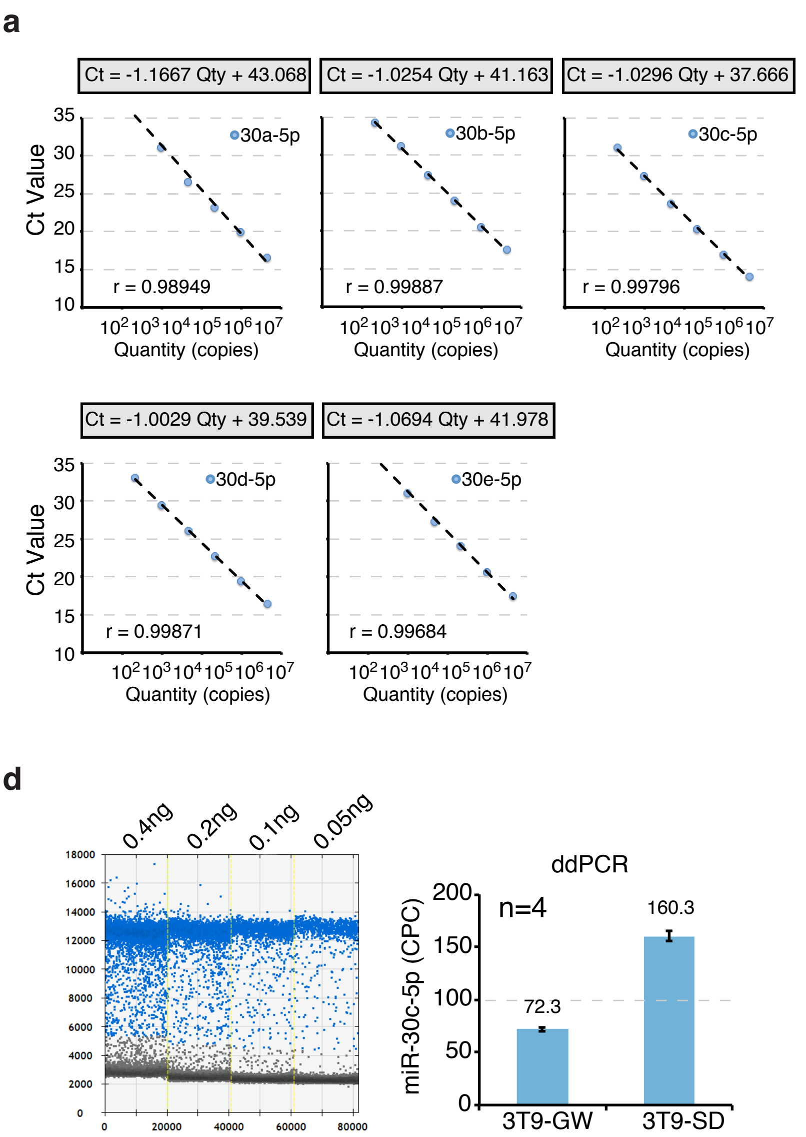

Supplementary Figure 3 - related to Figure 3. Absolute quantification of miR-30s and Serpine1 by Standard Curves and Droplet Digital PCR (ddPCR).

(a) Standard curves for the absolute quantification of miR-30s by serial dilutions of a cDNA obtained by spiking synthetic miRNAs in 3T9 total RNA [10^9 copies of the synthetic miRNA, reverse transcribed with the miScript system (Qiagen) and serially diluted for the qPCR reaction from 10^7 to 10^2 copies]. Results were fitted into linear correlation. Coefficient of determination (r) of fitting and linear equation is reported. (b) The specificty of miRNA detection (% of total) is reported for all miR-30s, as determined by using a synthetic spike-in and probing the cDNA with different miRNA assay (miScript system) of the 30 family by RT-qPCR. (c) Standard curves for the absolute quantification of Serpine1. Serial dilutions of a plasmid were used (AdEasy Shuttle vector), where Serpine1 3'UTR was cloned downstream of mCherry protein. Results were fitted into linear correlation. Coefficient of determination (r) of fitting and linear equation is reported. (d) Absolute quantification of miR-30c-5p was determined with an independent approach, namely using Droplet Digital PCR (QX200, Bio-Rad), with the Exiqon system. Left panel: droplet distribution using different amounts of cDNA as template is shown. Right panel: quantification of miR-30c-5p in growing (GW) and serum depleted 3T9 fibroblasts. Bar graph shows the average and the s.e.m. of 4 observations.

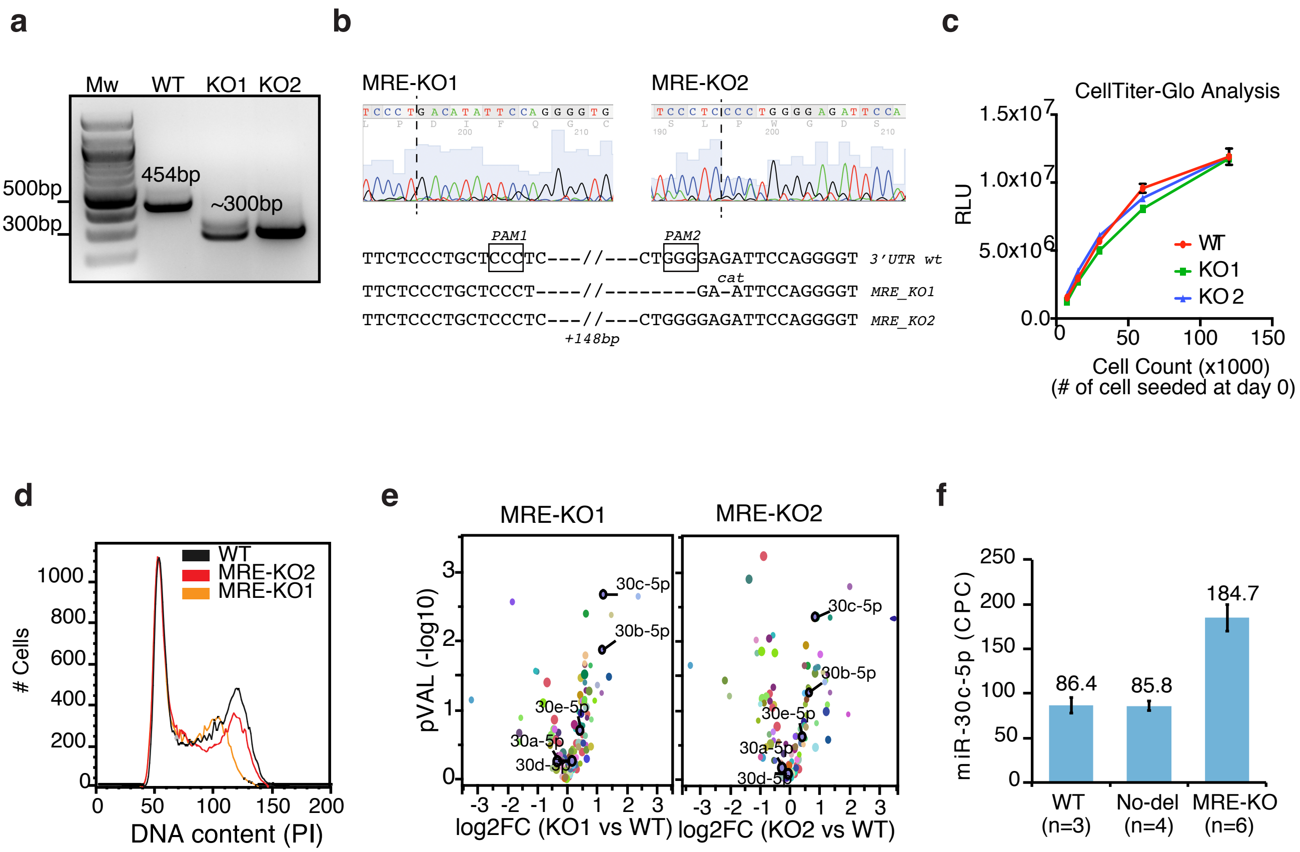

Supplementary Figure 4 - related to Figure 3. Characterization of MRE-KO clones.
(**a**) Genomic PCR analysis of the *Serpine1* locus was used to screen for MRE-KO clones. A region encompassing the deletion was amplified, producing either a 454 bp band (wild-type cells) or a shorter (~300 bp) PCR products (mutants clones). Two independent MRE-KO clones were identified and shown in the figure. (**b**) PCR-products (obtained as in **a**) were sequenced to confirm deletions at the targeted locus. Representative chromatograms (top) and sequences (bottom) showing the expected deletion in the *Serpine1* locus (dashed lines mark deletion junctions). (**c**) Cell viability of 3T9 WT and MRE-KO clones was evaluated by measuring ATP amount by CellTiter-Glo assay (Promega). Different cell numbers were plated in triplicate in a MW24 (x-axis). Luminescence (RLU, y-axis) was read after 24hrs. No significant differences were found. **(d)** Cell cycle profile of asynchronously growing WT and MRE-KO cells was performed with propidium iodide staining by FACS. (**e**) Small RNA sequencing was performed to measure miRNA expression in WT (n=4) and bi-allelic MRE-KO clones (KO1 and KO2; n=3 each) in asynchronously growing conditions. The graphs show the log2 fold changes (x-axis) and pValue (y-axis) of each MRE-KO clone vs WT cells. Members of the miR-30 family were highlighted. (**f**) Absolute quantification of miR-30c-5p in wild-type (WT) cells, negative clones (no-del) and MRE-KO clones by RT-qPCR. No-del clones are clones that has been subjected to transfection with sgRNA+CRISPR/Cas9 but are not bearing the deletion at Serpine1 locus. Bar graph shows the average and the s.e.m. of independent observations. Number of replicates is also reported.

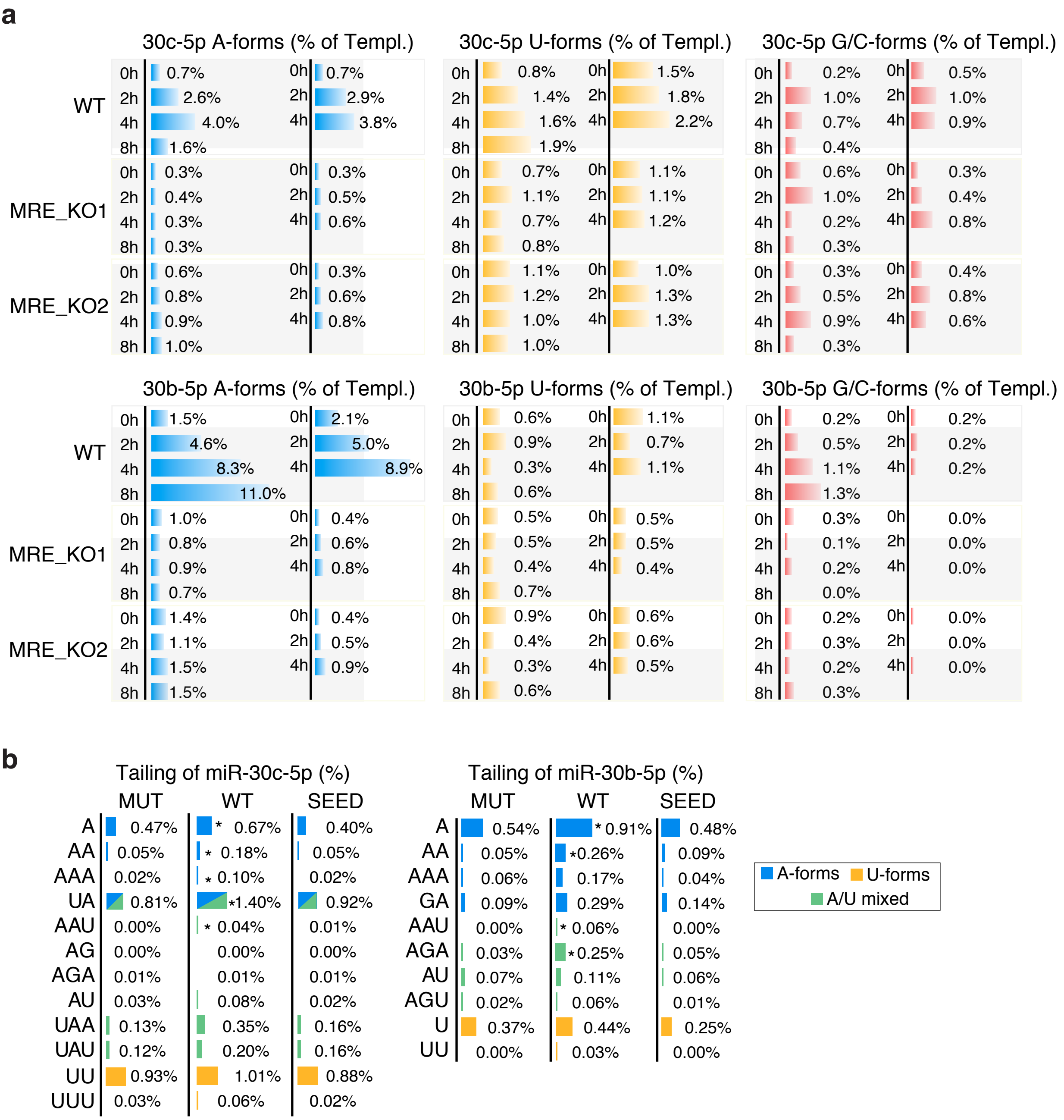

Supplementary Figure 5 - related to Figure 3 and Figure 4. Details on tailing isoforms in MRE-KO clones.
(**a**) Bar plots showing the percentage of isomiRs for miR-30c-5p (upper panel) or miR-30b-5p (lower) in quiescent 3T9 cells and during cell cycle re-entry by serum stimulation. Isforms were measured at different time points by small RNA sequencing in parental cells (WT) and MRE-KO clones. The graph reports the percentage of tailed isoforms distinguished into groups by the type of modification at the miRNA 3' end (Adenylation: "A-forms"; Uridylation: "U-forms"; others: "G/C-forms"). Results are reported as percentage of templated ("Templ.") reads (i.e. the sum of canonical miRNA and other templated isoforms). Two independent biological experiments are shown side by side. (**b**) bar plots showing the most frequent isomiRs for miR-30c-5p (left) or miR-30b-5 (right) in asynchronously growing MRE-KO cells expressing the wild type form of the 3'UTR of Serpine1 (WT) and two mutated 3'UTR, in which the miR-30 MRE was either completely abrogated (MUT) or mutated only in the 3'compensatory site, keeping an intact seed sequence (SEED). An asterisk marks significantly different isoforms (Student's test, $p<0.05$).

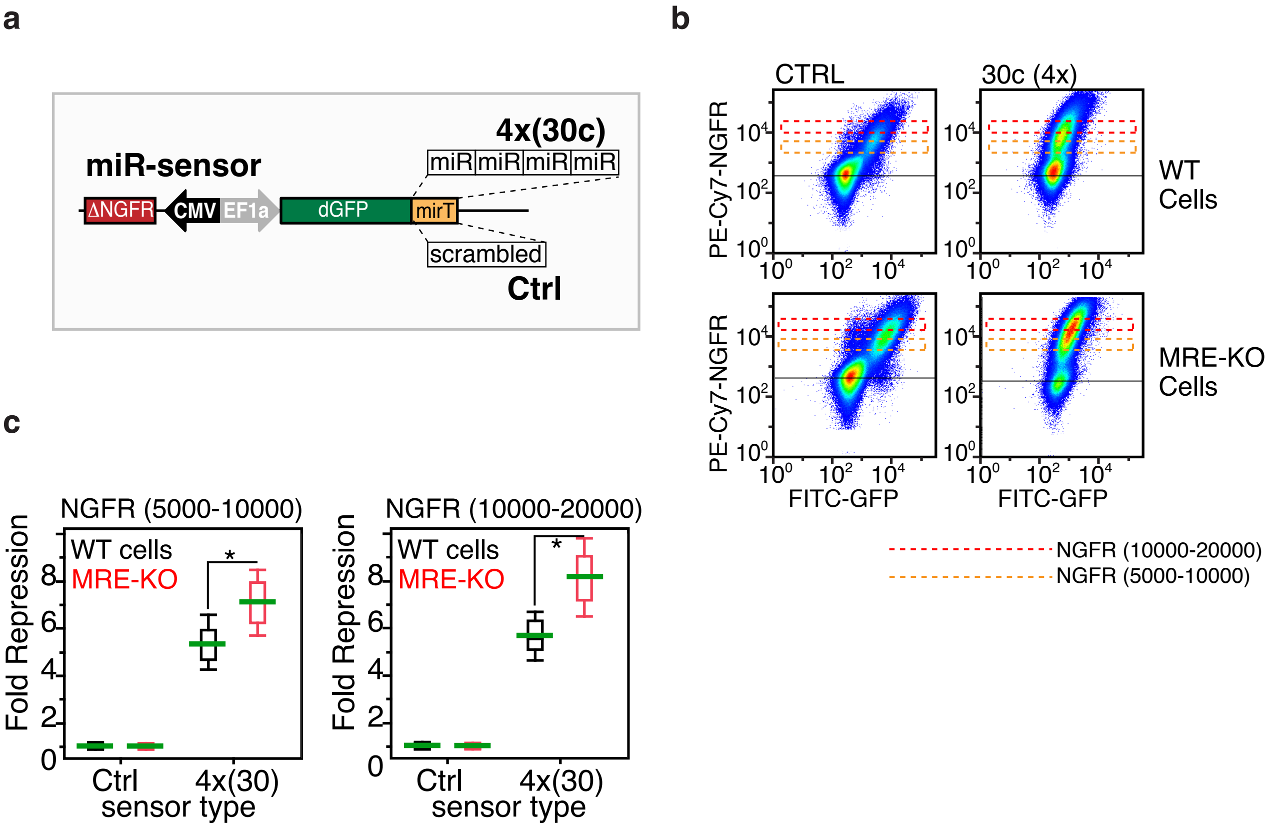

Supplementary Figure 6 - related to Figure 5. MiR-30c activity measured by miR-sensor. (**a**) Scheme of the miR-sensor, a lentiviral bidirectional reporter used to monitor miRNA activity by GFP levels. Two constructs were used, one with four repeats of miR-30c (4x30c) in the 3′ UTR of a destabilized GFP (dGFP), and one with an unrelated sequence used as control (Ctrl). (**b**) Representative FACS sorting of cells transduced with the lentiviral miR-sensor. Shown is the profile of GFP (miRNA responsive) and ΔNGFR (used to normalize transduction efficiency) of WT and MRE-KO cells infected with lentiviral reporter for miR-30c-5p activity (30c) or a negative control (CTRL). Red and orange boxes show the bins of ΔNGFR that were considered for the analyses reported in Panel C. (**c**) WT 3T9 cells or MRE-KO cells were infected with the reporter. The repression of dGFP levels was measured by FACS in WT and MRE-KO cells using two intervals of ΔNGFR expression, which normalizes for transduction efficiency (low, 5000– 10,000, or moderate, 10,000-20,000). Fold repression (dGFPcontrol / d GFP30c) was calculated over ten bins (each comprising 1000 cells). Asterisks mark significant values (*$p < 0.01$, Wilcoxon test).

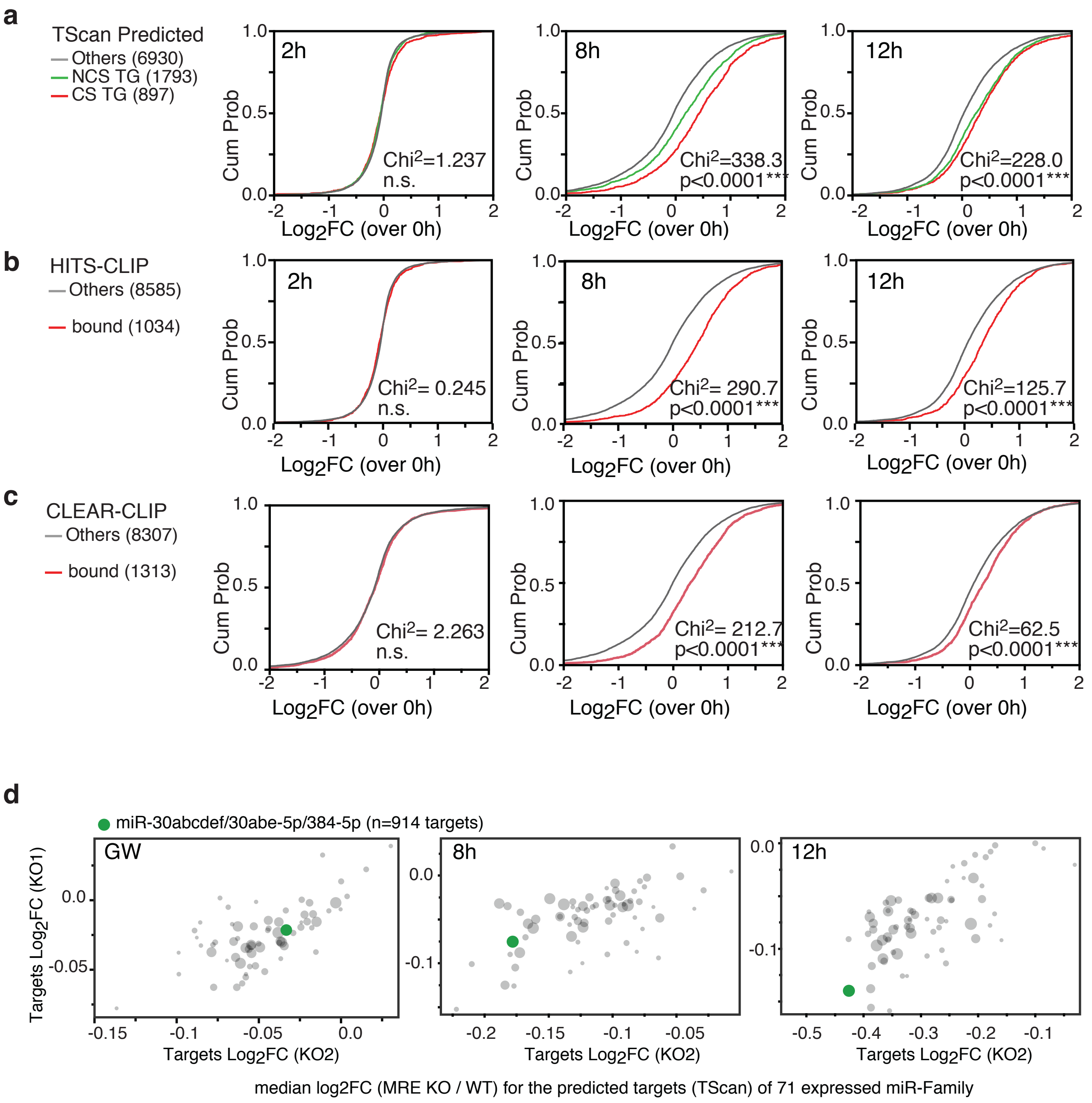

Supplementary Figure 7 - related to Figure 5. MiR-30c activity on targets
(**a-c**). Analysis of the dynamics of targets upon serum stimulation in wild-type 3T9 cells. Cumulative distribution functions (CDF) of log2FC (over quiescent cells) are shown for different groups of targets: (**a**) predicted mir-30 targets [TargetScan7.1: conserved (CS) or not conserved (NCS)]; (**b,c**) validated miR-30 targets (HITS-CLIP or CLEAR-CLIP). Statistical analyses (chi-square and p-value) were performed by Wilcoxon test. (**d**) Predicted targets for all expressed miRNA families (n=71) were identified by TargetScan7.1. The bubble plots report the median log2 fold changes of each MRE-KO clone vs WT cells, calculated for every miRNA family having at least 100 expressed targets. miR-30 family is highlighted in green. Bubble size is proportional to the number of targets considered for each family.

Supplementary Table 1 I Primers used for RT-qPCR

mouse genes	FORWARD	REVERSE
pri-let7b-let7c2	TGGGGCATAGCGATGAAAAC	TGTGAGCACAGGTCTGATATGG
pri-miR-155	AAGGCCTGTTACTAGCACTCAC	AGGGTGACTCTTGGACTTGTC
pri- miR-30b-30d	ATCACCATGCCCAGCTAAAG	TTCTGAATCAAAATATTGGTATCATCT
pri-miR-30a/30c2	GCCCCTGTTCTAACACGTCA	AGCAGCACAAGCATCAGGAT
pri-miR-30e/30c1	ATTGCAGGTTTGTGCCCATG	AGCTCCAAACGAAGAGAGACAG
Rplp0	TTCATTGTGGGAGCAGAC	CAGCAGTTTCTCCAGAGC
Serpine1 (3'UTR)	CAGAAGAGGGAAAAGGGGCT	CTCACATACAGCAGCCGGAA
Serpine1(3'UTR-del)	AAATGGTGGCCCAATAGCG	AAGCAAGCTGTGTCAAGGG

Supplementary Table 2 l Oligonucleotides that were used to clone Serpine1 MRE mutant.

oligo name	sequence
serpine1_WT_FW	AATTCTTAATTAAGCTTGCTTATGTTATTTCAGAGTGTAGGTGACTTGTTTACACAGCTTTTTTCGACCCACAAC
serpine1_m4_FW	AATTCTTAATTAAGCTTGCTTATGTTATTCCGGAGTGTAGGTGACTTGTTTACACAGCTTTTTTCGACCCACAAC
serpine1_m5_FW	AATTCTTAATTAAGCTTGCTTATGTTATTCCGCAGTGTAGGTGACTTGTTTACACAGCTTTTTTCGACCCACAAC
serpine1_m6_FW	AATTCTTAATTAAGCTTGCTTATGTTATTCCGCCGTGTAGGTGACTTGTTTACACAGCTTTTTTCGACCCACAAC
serpine1_b7_FW	AATTCTTAATTAAGCTTGCTTATGTTATTTCAGAGTGTAACTGACTTGTTTACACAGCTTTTTTCGACCCACAAC
serpine1_WT_REV	TCGAGTTGTGGGTCGAAAAAAGCTGTGTAAACAAGTCACCTACACTCTGAAATAACATAAGCAAGCTTAATTAAG
serpine1_m4_REV	TCGAGTTGTGGGTCGAAAAAAGCTGTGTAAACAAGTCACCTACACTCCGGAATAACATAAGCAAGCTTAATTAAG
serpine1_m5_REV	TCGAGTTGTGGGTCGAAAAAAGCTGTGTAAACAAGTCACCTACACTGCGGAATAACATAAGCAAGCTTAATTAAG
serpine1_m6_REV	TCGAGTTGTGGGTCGAAAAAAGCTGTGTAAACAAGTCACCTACACGGCGGAATAACATAAGCAAGCTTAATTAAG
serpine1_b7_REV	TCGAGTTGTGGGTCGAAAAAAGCTGTGTAAACAAGTCAGTTACACTCTGAAATAACATAAGCAAGCTTAATTAAG
