# Supplementary material for: Endogenous transcripts control miRNA levels and activity in mammalian cells by target-directed miRNA degradation
Source: Nat Commun. 2018 Aug 7;9:3119. doi: 10.1038/s41467-018-05182-9 (PMC6081425; doi:10.1038/s41467-018-05182-9)
Supplement: Supplementary file 3 — Description of Additional Supplementary Files [file 41467_2018_5182_MOESM3_ESM.pdf]

Description of Additional Supplementary Files

File Name: Supplementary Movie 1
Description: Representative movie of time-lapse microscopy for parental 3T9 cells upon serum stimulation. Mitosis and cell deaths were manually annotated at each time frame and reported in Fig. 6g,h.

File Name: Supplementary Movie 2
Description: Representative movie of time-lapse microscopy for MRE KO1 upon serum stimulation. Mitosis and cell deaths were manually annotated at each time frame and reported in Fig. 6g,h.

File Name: Supplementary Movie 3
Description: Representative movie of time-lapse microscopy for MRE KO2 upon serum stimulation. Mitosis and cell deaths were manually annotated at each time frame and reported in Fig. 6g,h.

File Name: Supplementary Data 1
Description: List of candidate TDMD Targets in 3T9 Fibroblasts, related to Fig.1. Two datasheets: a) "miRNA_target pairs 3T9 datasheet", showing the complete list of 18856 target:miRNA pairs expressed in 3T9 serum model. The columns marked in red contain the data reported in Fig. 1d; b) "3C target distribution datasheet" reports for each miRNA the % of 3C target contribution over the total number of targets.

File Name: Supplementary Data 2
Description: Small RNA sequencing data for parental 3T9 cells, MRE KO1 and MRE KO2 in growing conditions. The datasheet contains reads per million (RPM) values for all expressed miRNAs in each replica, as well as the log2 fold change (Log2FC) and pvalues (-log10, Student's T-test) of MRE KO cells compared to parental 3T9.

File Name: Supplementary Data 3
Description: RNA sequencing data for parental 3T9 cells, MRE KO1 and MRE KO2. Three datasheets: a) "RNAseq_data", showing expression levels (as transcripts per million, TPM) and regulation (as log2 fold change MRE KO vs. WT) for all expressed genes (n=9619); b) "244 DEGs in GW", containing information of the differentially regulated genes in growing conditions, related to Fig.7; c) "1648_DEGs timecourse", containing information of the differentially regulated genes in time course experiment, related to Fig 7.
